# Phytochemicals as modulators of M1-M2 macrophages in inflammation

**DOI:** 10.18632/oncotarget.24788

**Published:** 2018-04-03

**Authors:** Uzma Saqib, Sutripta Sarkar, Kyoungho Suk, Owais Mohammad, Mirza S. Baig, Rajkumar Savai

**Affiliations:** ^1^ Discipline of Chemistry, School of Basic Sciences, Indian Institute of Technology (IIT) Indore, MP, India; ^2^ PostGraduate Department of Food & Nutrition, BRSN College (affiliated to WBSU), Kolkata, WB, India; ^3^ Department of Pharmacology, Kyungpook National University School of Medicine, Joong-gu Daegu, South Korea; ^4^ Interdisciplinary Biotechnology Unit, Aligarh Muslim University (AMU), Aligarh, UP, India; ^5^ Discipline of Biosciences and Biomedical Engineering (BSBE), Indian Institute of Technology (IIT), Indore, MP, India; ^6^ Department of Internal Medicine, Universities of Giessen and Marburg Lung Center (UGMLC), member of the German Center for Lung Research (DZL), Justus Liebig University, Giessen 35392, Germany; ^7^ Max Planck Institute for Heart and Lung Research, Department of Lung Development and Remodeling, Member of the DZL, Bad Nauheim, Germany

**Keywords:** Anti-inflammatory cytokines, inflammation, M1–M2 macrophages, natural compounds, pro-inflammatory cytokines

## Abstract

Macrophages are critical mediators of the innate immune response against foreign pathogens, including bacteria, physical stress, and injury. Therefore, these cells play a key role in the “inflammatory pathway” which in turn can lead to an array of diseases and disorders such as autoimmune neuropathies and myocarditis, inflammatory bowel disease, atherosclerosis, sepsis, arthritis, diabetes, and angiogenesis. Recently, more studies have focused on the macrophages inflammatory diseases since the discovery of the two subtypes of macrophages, which are differentiated on the basis of their phenotype and distinct gene expression pattern. Of these, M1 macrophages are pro-inflammatory and responsible for inflammatory signaling, while M2 are anti-inflammatory macrophages that participate in the resolution of the inflammatory process, M2 macrophages produce anti-inflammatory cytokines, thereby contributing to tissue healing. Many studies have shown the role of these two subtypes in the inflammatory pathway, and their emergence appears to decide the fate of inflammatory signaling and disease progression. As a next step in directing the pro-inflammatory response toward the anti-inflammatory type after an insult by a foreign pathogen (e. g., bacterial lipopolysaccharide), investigators have identified many natural compounds that have the potential to modulate M1 to M2 macrophages. In this review, we provide a focused discussion of advances in the identification of natural therapeutic molecules with anti-inflammatory properties that modulate the phenotype of macrophages from M1 to M2.

## INTRODUCTION

**Figure 1:**
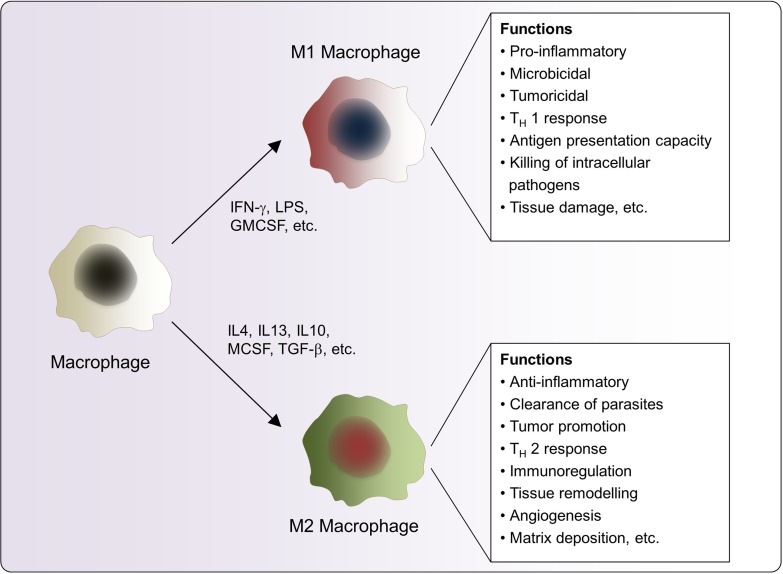
Macrophage polarization and specific functions of M1 and M2 macrophages Different stimuli activate the generation of M1 and M2 macrophages. M1, M2 macrophages differ phenotypically as well as in their release of pro-and anti-inflammatory cytokines respectively.

Macrophages form an essential component of innate immunity by inhibiting or promoting cellular proliferation and tissue repair [1]. They are highly plastic and dynamic in nature, which has been attributed to their ease in adapting alternate phenotypes in response to various external stimuli [2]. Macrophages are distinctly subdivided into the classical M1 and alternative M2 categories, which in turn correspond to the Th1–Th2 polarization of T cells respectively (Figure 1). This process represents the extremes of the dynamic changing state of macrophage activation [2]. Pro-inflammatory M1-macrophages release cytokines that inhibit the proliferation of malignant cells [3] and counter various pathogens [4]. In contrast, M2-macrophages or tumor-associated macrophages (TAM’s) release cytokines that promote tumor growth and dissemination along with tissue repair [5]. The M1–M2 macrophage polarization process is tightly regulated by key signaling events. The classical activation of macrophages occurs following an injury or infection by agents such as microbial products or pro-inflammatory cytokines including bacterial lipopolysaccharides (LPS), interferon-γ (IFN-γ) or tumor necrosis factor-α (TNF-α) [6]. M1 macrophages are characterized by the production of pro-inflammatory cytokines, the release of interleukin (IL)-12 and IL-23, and high levels of reactive oxygen intermediates (ROIs) and nitric oxide (NO). By contrast, M2 macrophages are activated by entirely different stimuli and are observed in the healing phase without the prevalence of infection [7]. These stimuli include IL-4 and/or IL-13, immune complexes and toll-like receptor (TLR), IL-1 receptor ligands, and IL-10. They are further characterized by the secretion of anti-inflammatory cytokines such asIL-10, chemokine (C-C motif) ligands (CCL)18 and CCL22, and the upregulation of dectin-1, mannose receptor CD206 (MRC1), scavenger receptor A, scavenger receptor B-1, CD163, C-C chemokine receptor type 2 (CCR2), C–X–C motif chemokine receptor (CXCR) 1,CXCR2 and dendritic cell-specific intercellular adhesion molecule-3-grabbing non-integrin (DC-SIGN) [8–9]. Moreover, M2 macrophages produce ornithine and polyamines through the arginase pathway [10] while M1 macrophages generate hazardous NO or ROI. To summarize the molecular agents released by classical M1 macrophages, pro-inflammatory cytokines such as TNF-α, IL-1, IL-6, IL-12, Type I IFN, C–X–C motif chemokine ligand (CXCL) 1–3, CXCL-5, and CXCL8–10 form the major pool [11]. On the other hand, the alternative M2 macrophages generate an array of anti-inflammatory cytokine such as IL-10, IL-4 and very low levels of pro-inflammatory cytokines such as IL-12 among others [12]. An optimum balance between M1 and M2 macrophage is very important at the basal, as well as advanced level, of immune regulation as any imbalance in the two states would be expected to cause the dysregulation of the immune pathway. The plasticity of macrophage transition might be attributed to the complex signaling pathways associated with the two phenotypes. As multiple molecules common to both phenotypes are involved in the transition, it could be well understood that the interconversion is mostly due to the effect of one subset on the other and vice versa. This could be best exemplified by the studies of Fernando *et al.* [13] who showed that a pro-Inflammatory cytokine, IL-6 enhances the polarization of M2 macrophages. They showed that IL-6 reinforced the IL-4+IL-13 polarization of macrophages into the M2 subtype diverted them for a range of additional immune-regulatory roles. Further, several studies have shown that the M2 phenotype can also be enhanced by various cytokines including IL-33, along with interactions with fibroblasts and regulatory T cells [14–16]. IL-6 has also been shown to promote the differentiation of pro-inflammatory IL-17-producing Th17 cells along with suppressing the production of FoxP3^+^ regulatory T cells (Treg) [17]. Thus transitions between the M1-M2 phenotypes have been influence by multiple factors and pathways and a detailed understanding of these are still underway.

The opposing effects of macrophages in the innate immunological response have been a deep subject of study by various investigators around the world. Indeed, there has been much emphasis on the identification of molecules associated with the transition of M1–M2 phenotypes. Among the latest macrophage-mediated inflammatory therapeutic strategies, various efforts to modulate the signaling from pro-inflammatory M1 to anti-inflammatory M2 have been adopted [18]. Many natural and synthetic pharmacological agents that modulate the inflammatory pathway from M1 to M2 have been identified [19–20]. Recently, there have been several studies in which investigators have successfully shown that the inflammatory pathway can be diverted from a pro-inflammatory to an anti-inflammatory path by utilizing the pharmacological application of various natural analogs [19]. The actual mechanism of action of these molecules that serves to modulate the M1–M2 phenotype remains unknown. However, with the discovery of more molecules and signaling pathways associated with M1–M2 macrophage state, there is a hope for a clearer understanding of the actual mechanisms of action of these natural compounds. In the current review, we discuss the majority of the well-known natural pharmacological agents that have been discovered during the past decade or longer that have shown to impact the phenotype transition from M1 to M2 thereby acting as potent anti-inflammatory agents. Supplementary Table 1 summarizes important pharmacological modulators of M1-M2 polarization. To the best of our knowledge, this review is first to highlight the importance of natural compounds as potent M1–M2 modulators.

## NATURAL PHARMACOLOGICAL MODULATORS

### Lupeol

Lupeol (or Fagarsterol) is a pentacyclic triterpenoid secondary metabolite prominently found in fruit plants such as olive, mango, strawberry and grapes, as well as in vegetables such as white cabbage, pepper, cucumber, tomato [21]. Lupeol and its derivatives (linoleate, acetate and palmitate) are known to have many biological activities including anti-inflammatory and anti-arthritic effects [22]. It is assumed to be a multi target agent with beneficial activity against inflammation, cancer, arthritis, diabetes, heart diseases, renal, and hepatic toxicity [23]. Research into lupeol as a potent compound dates to more than a decade back, when Geetha *et al.* [24] were among the first to show the anti-inflammatory activity of lupeol and its derivatives in rats. Many studies [25] have since demonstrated that lupeol possesses anti-inflammatory activity on cytotoxic and helper T cells by inhibiting the production of cytokines like TNF-α and IL-2, and IFN-ᵞ. Other investigators around the world have confirmed the ability of this compound to be an anti-inflammatory agent [22]. The potent biological activities of lupeol have been discussed in detail in multiple reviews [21, 23, 26]. Among the detailed studies on the anti-inflammatory properties of lupeol, those presented by Kang *et al.* [27] are very promising. They showed that lupeol inhibits Latent membrane protein 1(LMP1)-induced NF-κB activation and reduces NF-κB-dependent lymphoblastoid cell line viability. The anti-pro-inflammatory cytokine release activity of lupeol has been previously reported in M1 macrophages [28], however, the actual M1 to M2 phenotypic switch has only been recently demonstrated [29]. They showed that lupeol switches M1 to M2 macrophages and ameliorates experimental inflammatory bowel disease. In their studies, LPS-treated M1 andM2 macrophages were treated with lupeol which resulted in a marked decrease in the production of pro-inflammatory cytokines; IL-12, IL6, IL-1β, TNF-α along with an increased production of the anti-inflammatory cytokine IL-10. These studies indicates the potency of lupeol in ameliorating experimental inflammatory bowel disease as well as other inflammatory diseases through the inhibition of M1 macrophages and the promotion M2 macrophages.

### Stilbenes

#### Resveratrol

Resveratrol (3,5,4′-trihydroxystilbene) is a natural polyphenolic phytoalexin. It belongs to the stilbene family and is present in more than 70 plant species including berries, peanuts and grapes [30]. Resveratrol has been shown to have potent antioxidant activity by reducing the generation of mitochondrial reactive oxygen species (ROS) and promoting mitochondrial biogenesis [31]. Resveratrol has also demonstrated therapeutic potential in a myriad of disease models due to its ability to modulate numerous signaling pathways. These include the inhibition of vasodilation [32] and platelet aggregation in atherosclerosis [33], cardio protection [34], chemoprevention [35], and the protection against stress, neurodegeneration and other anti-inflammatory diseases [36]. The diverse beneficial effects of resveratrol are believed to be primarily driven by modulations of important proteins such as transthyretin, cardiac regulatory protein troponin C, sulfotransfase1B1, quinone reductase 2, peroxisome proliferator-activated receptor gamma (PPAR-γ), concanavalin A, sirtuin (SIRT1, 3 and 5) and transcriptional factors such as NFkB [37–39].

Buttari *et al.* [40] have clearly demonstrated the ability of resveratrol to counteract 7-oxo-cholesterol-triggered pro-inflammatory signaling in macrophages. They demonstrated that in the M1 subset, resveratrol prevented the downregulation of CD16 and the upregulation of matrix metalloproteinase-2 (MMP-2) in response to 7-oxo-cholesterol, whereas in M2 macrophages it prevented the upregulation of CD14, MMP-2, and MMP-9 and the downregulation of endocytosis. Further, resveratrol prevented the upregulation of several pro-inflammatory and proangiogenic molecules in both subsets. Resveratrol thus reversed the oxysterol-induced switch of the M2-M1 phenotype. Similar studies have supported this observation demonstrating that resveratrol increasesM2 phenotype marker; arginase and decreasesM1 phenotype markers, including inducible nitric oxide synthase (iNOS) and NO production in LPS-treated RAW264.7 cells, by a possible catecholamine mediated mechanism [41].

#### Malibatol A

Malibatol A (MA) is an oligostilbene isolated from the organic extract of the leaves of *Hopeamalibato*. MA is an oligomer of the famous compound resveratrol and has also shown to have potent antioxidant properties [42]. Studies performed by Yang *et al.* [43], have shown that MA protects against brain injury by reversing mitochondrial dysfunction in experimental stroke and has a protective effect on brain damage after ischemia/reperfusion injury. The M1–M2 modulatory potential of MA has been recently described [44], that demonstrated that MA inhibited the expression of pro-inflammatory cytokines and M1 markers (CD16, CD32, and CD86) while increasing M2 markers (CD206, YM-1) in LPS-stimulated microglia. MA, also decreased the infarct size and alleviated brain injury after mice middle cerebral artery occlusion. This function of MA appears to be due to the activation of nuclear receptor PPAR-γ [44]. Collectively, the anti-inflammatory effects of MA that occur in a PPAR-γ-dependent manner, suggest it is a potential candidate for the stroke treatment as well as other inflammatory diseases via M1–M2 modulation.

### Geraniin

Geraniin is a polyphenolic compound of dehydroellagitannin specifically found in the Japanese medicinal plant, *Geranium thunbergii* [45]. Reports have suggested it is a novel heat shock protein (Hsp) 90 inhibitor for treating tumor growth [46]. Plant extracts of Geranium bellum and aqueous leaf extracts of *Phyllanthusmuellerianus* (Kuntze) have been shown to have anti-inflammatory activities with geraniin as the most potent compound [47]. Similarly, extracts of Geranium thunbergii significantly inhibit the LPS- and IFN-γ-induced expression of pro-inflammatory genes, such as iNOS, TNF-α and IL-1β [48] by a mechanism mediated by increased nuclear factor erythroid 2-related factor 2 (Nrf2) activity. Other studies using pure geraniin have validated these observations by showing that it is indeed geraniin that is responsible for the inhibition of LPS-induced inflammation by regulating NF-κB via the Nrf2 pathway in macrophages. Recently, Liu *et al.* [49] have published their latest findings describing the role of Geraniin in LPS-induced THP-1 macrophages by switching them to theM2 phenotype through the suppressor of cytokine signaling 1 (SOCS1)/NF-κB pathway. In this study, geraniin downregulated LPS-induced M1 macrophage pro-inflammatory cytokines; including TNF-α and IL-6, and the production of ROS and NO, as well as iNOS activity in THP-1 macrophages. Further, geraniin upregulated the expression of SOCS1, an upstream regulator of NF-κB activation that can directly bind to NF-κB-p65 and downregulate it, thus inhibiting NF-κB activation. These studies define the promising role of geraniin in M1-M2 macrophage polarization via SOCS1 upregulation and indicate the compound may be a highly effective anti-inflammatory therapeutic agent.

### Compound A

Compound A (CpdA) is derived from the Namibian shrub *Salsolatuberculatiformis Botschantzev* [50]. This aziridine precursor was initially identified as a glucocorticoid receptor (GR) activator, capable of efficiently down modulating NF-κB-driven genes [51]. CpdA also has been shown to inhibit the production of fluticasone-resistant chemokines CCL5, CX3CL1, and CXCL10 in human airway smooth muscle (ASM) cells [50]. Moreover, CpdA enhances Hsp70 gene promoter activation by reducing TNF-stimulated IκBα degradation and NF-κB p65 nuclear translocation [52]. It exerts an anti-inflammatory effect by down modulating TNF-α induced pro-inflammatory gene expression, such as IL-6 and E-selectin as well as interfering with the DNA-binding capacity of NF-κB by directly inhibiting the transactivation capacity of the NF-κB p65 subunit via activated GR [51]. The anti-inflammatory mechanism of CpdA appears to involve both a reduction of the *in vivo* DNA-binding activity of p65 as well as an interference with the transactivation potential of NF-κB [53]. CpdA also attenuates collagen-induced arthritis (CIA) [54]. The M1–M2 modulatory activity of CpdA against the progression of immunoinflammatory diabetes has been shown to occur via the conversion of the pro-inflammatory M1/Th1/Th17 phenotype to anti-inflammatory M2/Th2/Treg phenotype [55]. The CpdA-induced switch of macrophages from M1 to M2, has recently been shown in an experimental autoimmune neuritis model where CpdA depressed Th1 and Th17 cytokines and increased Th2 cytokine and Foxp3 expression [56]. Thus, the immuno-modulatory roles of CpdA in diabetes, autoimmune neuropathies and other diseases warrant further investigation of its potential to mitigate other related inflammatory disorders via M1–M2 modulation.

### CP-25

Although the pharmacological properties of paeoniflorin (Pae) have been known for several years, those of its acylated derivative, CP-25 (paeoniflorin-6′-O-benzene sulfonate) have only recently been identified [57]. A recent study has demonstrated the M1–M2 macrophage modulating potential of CP-25 [58], in whichCP-25-treated rats exhibited decreased pro-inflammatory cytokines (IL-1β, IL-6, IL-17 and TNF-α) coupled with an increase in the anti-inflammatory cytokine TGF-β1. Similarly, the application of CP-25 in a rat adjuvant-induced arthritis model of humanRA29 shows an anti-arthritic activity by suppressing inflammation and bone damage primarily by modulating inflammatory mediators like Th17-IL-17. Hence, CP-25 appears to play an important role in modulating M1–M2 phenotype and may be a good starting point for pharmaceutical development against human rheumatoid arthritis.

### Aloe-emodin

Aloe-emodin (AE) is a major anthraquinone present in the aloe plant [59]. AE has been shown to dose dependently inhibit the levels of NO and prostaglandin E2 (PGE2) by blocking the mRNA expression of iNOS and cyclooxygenase-2 (COX-2) in LPS-stimulated macrophages [60]. AE also ameliorated lung injury via the inhibition of pro-inflammatory cytokine production and the p38 mitogen-activated protein kinase (MAPK) pathway in an animal model [61]. Moreover, AE inhibits the NF-κB/IFN regulatory factor 5 (IRF5)/signal transducer activator of transcription 1 (STAT1) and IRF4/STAT6 signaling pathways [62]. Although fewer studies have investigated the biological activity and exact mechanism of action of AE, its anti-inflammatory activity has been demonstrated by dose-dependently inhibiting iNOS mRNA expression and NO production [63–65], making it a potent M1–M2 modulator.

### Flavonoids

#### Quercetin

Quercetin is a flavonol found in many fruits, vegetables, leaves, and grains. It has been shown to modify the phenotype ratio of M1–M2 macrophages [66]. It lowers the pro-inflammatory cytokine levels via enhancing adenosine monophosphate-activated protein kinase α1 phosphorylation and SIRT1 expression in epididymis adipose tissues (EATs) [67]. Similar effects have been shown by Kim and Park [68] on double-stranded RNA-induced mouse macrophages; quercetin significantly inhibited the production of pro-inflammatory mediators such as NO, IL-6, monocyte chemotactic protein 1(MCP-1), and IFN-γ-induced protein 10 (IP-10) and regulated the activation of normal T-cell expression and secretion, granulocyte-macrophage colony-stimulating factor (GM-CSF), granulocyte-colony stimulating factor (G-CSF), TNF-α, leukemia inhibitory factor, CXCL5, vascular endothelial growth factor (VEGF), STAT1 and 3. Quercetin, along with lycopene and tyrosol also inhibits IFN-γ, iNOS and COX-2 gene expression and NF-κB, IRF-1, and STAT-1α activation induced by ROS [69]. Hämäläinen *et al.* have also characterized the effects of many flavonoids on PGE2 production along with COX-2 and microsomal prostaglandin E synthase-1 (mPGES-1) expression in activated macrophages. As many as 12 flavonoids including flavone, luteolin-7-glucoside, kaempferol, isorhamnetin, morin, quercetin, naringenin, taxifolin, pelargonidin, daidzein, genistein, and genistin were able to effectively inhibit LPS-induced PGE2 production [70]. The atheroprotective properties of quercetin appear to be due to its interference with proatherogenic activities of macrophages such as foam cell formation and pro-inflammatory responses [71]. These properties may be due to the mechanism involving the upregulation of the expression of PPARγ and ATP-binding cassette transporter (ABCA1), which has been as shown in THP-1 cells [72]. Quercetin has also been shown to attenuate the basal expression of inflammatory genes including TNF-α, IL-6, IL-8, IL-1β, IP10, COX-2, phosphorylated c-Jun N-terminal kinase (JNK), c-Jun, and IκBα degradation in macrophages [73]. Many other studies have shown a direct role of quercetin in modulating the M1 to M2 phenotype in various models [68–70].

#### Curcumin

Curcumin is a yellow pigment from the famous Asian medicinal plant *Curcuma longa* commonly called turmeric. In ancient Ayurvedic medicine, curcumin has been regarded as a pharmaceutical agent for many pathological conditions [74] and the beneficial effects of curcumin as an anti-cancer and anti-inflammatory compound have long been reported [75]. Multiple reviews have covered the pharmaceutical properties of curcumin in various diseases [76–78]. Moreover, the effect of curcumin on the inhibition of pro-inflammatory cytokine secretion has been reported by Abe Y *et al.* [79], who clearly showed that curcumin inhibited the production of IL-8, macrophage inflammatory protein-1 alpha (MIP-1α), MCP-1, IL-1β, and TNF-α by LPS-stimulated monocytes as well as alveolar macrophages (AMs). Curcumin also has the potential to enhance the secretion of M2 macrophage markers such as the macrophage mannose receptor (MMR), Arg-1, PPAR-γ, IL-4 and/or IL-13 in Raw264.7 macrophages. These effects have been observed in an experimental autoimmune myocarditis (EAM) model and hyaline membrane disease in which curcumin polarizes M0 and M1 macrophages to the M2 phenotype. The anti-inflammatory activity of curcumin in macrophages stimulated by LPS has also been reported and curcumin was shown to inhibit TNF-α and IL-1β expression [80]. Curcumin appears shown to have potent pharmacological properties for the treatment of tendon inflammation through the modulation of PI-3K/Akt-mediated NF-κB signaling [81]. Furthermore, the anti-inflammatory activity of mono-carbonyl analogues of curcumin has also been observed in LPS-stimulated macrophages. [82]. Curcumin supplementation lowers TNF-α, IL-6, IL-8, and MCP-1 secretion in high glucose-treated cultured monocytes [83]. Moreover, curcumin has been shown to suppress the production of the pro-inflammatory cytokine IL-18 in LPS stimulated murine macrophage-like cells [84]. Numerous studies showing the potent pharmacological effect of curcumin in modulating M1–M2 macrophages makes it an extremely important natural anti-inflammatory agent.

#### Naringenin

The flavonoid, naringenin is predominantly found in grapefruit. The first reports on the macrophage modulatory role of naringen introduced it as a compound for further pharmacological investigation [85]. Naringenin has been shown to significantly inhibit the excessive production of NO and PGE2 in LPS treated macrophages and this inhibition is was associated with the downregulation of iNOS and COX-2 expression [86]. Naringenin also has been shown to attenuate the production of pro-inflammatory cytokines and chemokines, including IL-1β, TNF-α and MCP-1. Furthermore, naringenin-mediated attenuation of inflammation in BV2 cells is reportedly due to its suppression of the NF-κB p65 subunit translocation as well as phosphorylation of Akt and MAPKs [87]. Similar studies by Raza *et al.* (2013) have demonstrated that the neuroprotective effect of naringenin is mediated through the suppression of the NF-κB signaling pathway in experimental stroke [88]. The anti-neuroinflammatory role of naringenin is may be due to its ability to induce SOCS3 expression [89]. Dou *et al.* [90] showed that pre-administration of naringen significantly reduced the severity of colitis and resulted in downregulation of pro-inflammatory mediators such as iNOS, intercellular adhesion molecule-1 (ICAM-1), MCP-1, COX-2, TNF-α and IL-6 in the colon mucosa. In pancreatitis, naringenin reduced caspase-1 activity and the maturation of pro-inflammatory cytokines [91]. In another study, LPS-mediated DC maturation was effectively inhibited by naringenin as shown by reductions in the levels of pro-inflammatory cytokines/chemokines. Similar effects have been observed in a murine model of CIA, in which naringenin decreased LPS-induced MAPK and NF-κB signaling activation [92].

#### Apigenin

Apigenin (Api) is a naturally occurring plant flavonoid that is abundant in various fruits and vegetables. Api favors M2 polarization via PPARγ thereby blocking the inflammatory functions of adipose tissue macrophages [93]. It also plays a substantial role in suppressing obesity-related inflammation in animal models of obesity. Balex at al [94] showed that the anti-inflammatory effects of Api in LPS-mediated acute lung injury are due to its ability to inhibit COX-2 and NF-kB gene expression in the lung. A number of recent studies have highlighted the importance of Api as a potent M1–M2 modulator, downregulating the pro-inflammatory cytokine and NO production [95].

#### Chrysin

Chrysin (5,7-di-OH-flavone) is a widely distributed natural flavonoid, mostly present in the passion flowers of the *Passiflora* family. It has a similar action to Api discussed above, in attenuating inflammation by regulating M1/M2 status. Moreover, chrysin has been shown to promote an anti-inflammatory M2 phenotype alongside the inhibition of the M1 phenotype, in *in vitro* peritoneal and cultured macrophages via the activation of PPAR-γ [96].

#### Procyanidins

Procyanidins are oligomeric flavonoids found in plants such as apples, cinnamon, cocoa beans, and grapes. They have been shown to modulate the M1 stage of macrophages by suppressing the MAPK and NF-κB pathway, along with the inhibition of the production and secretion of inflammatory mediators [97].

### Epigallocatechin gallate

Epigallocatechin gallate (EGCG) is the major polyphenol present in green tea. It has been shown to inhibit the production of pro-inflammatory mediators including NO, and PGE2, by downregulating iNOS and COX-2 gene expression [98]. The anti-inflammatory properties of EGCG have recently been reviewed by Singh *et al.* [99].

### Berberine (BBR)

Berberine (BBR) is a natural alkaloid isolated from plants such as *Coptischinensis, Hydrastiscanadensis* and *Berberis vulgarism* and has long been used in Chinese medicines [100]. Many reports have indicated indicate the inhibitory effect of BBR on the production of pro-inflammatory cytokines in various cell lines including pancreatic *β*-cells, nerve cells, lung cells and rat kidney cells [101] and animal models of insulin resistance [102]. Moreover, BBR negatively regulates the NF-*κ*B signaling pathway; the anti-inflammatory properties of BBR may be attributed to its role the in downregulation of Th17 and Th1 cytokine secretion [103].

### Apocynin

Apocyninis a simple organic compound composed of a multi-substituted phenyl ring. It is isolated from *Picrorhizakurroa*, and is well known in traditional medicine for having potent antioxidant activity [104]. Apocynin demonstrates inhibitory effects on pro-inflammatory stimuli such as TNF-α, and LPS, along with Poly I:C induced activation of NF-κB and AP-1. Apocynin inhibits NF-κB activation induced by external stimuli thereby inhibiting the production of the pro-inflammatory cytokines, TNF-α, IL-1β and IL-6 [105]. Apocynin has also been clearly demonstrated to reduce lung inflammation [106].

### Paeonol

Paeonol (2′-hydroxy-4′-methoxyacetophenone) is a simple phenolic compound of Paeonia suffruticosa and has demonstrated anti-inflammatory properties. It has been shown to inhibit pro-inflammatory TNF-α and IL-1β production while enhancing anti-inflammatory IL-10 production in rat paw exudates after carrageenan injection [107]. Many other reports have discussed the anti-inflammatory activities of Paeonol is various disease models including neurodegenerative disorders and arthritis [108].

### Terpenes

#### Forskolin

Forskolin (*coleonol)* belongs to the labdane diterpene family and is an extract from the Indian coleus plant (*Coleus forskohli*). Although not many reports exist regarding its anti-inflammatory properties, it has been recently reported to strongly inhibit the LPS-induced increases in MCP-1, TLR-4, and NF*κ*B1 mRNA levels in adipocytes [109].

#### Triptolide

Triptolide (diterpenoid epoxide) has been used in traditional Chinese medicine and extracted from the thunder god vine, *Tripterygium wilfordii*. It has been shown to inhibit the LPS-induced expression of pro-inflammatory cytokines and chemokines such asIL-6, G-CSF, MCP-1, and IL-8 as well as ICAM-1 in human corneal fibroblasts [110]. Its synthetic derivative Minnelide has also been recently been shown to have anti-cancer properties [111].

#### Terpinen-4-ol

Terpinen-4-olis the main component of the essential oil of tea tree (*Melaleuca alternifolia*). It has been shown to suppress the production of inflammatory mediators such as TNF-α, IL-1β, IL-8, IL-10, and PGE2 after LPS stimulation in monocytes [112–113].

## SYNTHETIC AND MICROBIAL PRODUCT MODULATORS

In addition to the compounds discussed above, there have been a number of studies demonstrating the macrophage modulatory activity of synthetic compounds and microbial products. Although a detailed molecular mechanism for the M1–M2 switch is still unknown for many of them, a clear-cut role in the modulation has been well documented for most. One such synthetic modulator is Dexamethasone, a pregnane corticosteroid and a derivative of cortisol. It has been shown to induce macrophages to a predominantly M2 phenotype in Immune thrombocytopenia [114]. Fucoidan is a sulfated polysaccharide obtained from brown algae and seaweeds. Its anti-inflammatory effects on the LPS-induced production of pro-inflammatory mediators such as NO, PGE2, iNOS, COX-2, MCP-1, IL-1β and TNF-α in BV2 microglia have recently been observed [115]. BIO (6-bromoindirubin-3′-oxime) has also been identified as a synthetic derivative of the natural compound 6-bromoindirubin produced by the Mediterranean mollusk *Hexaplextrunculus.* It was initially identified as a glycogen synthase kinase-3 (GSK-3) inhibitor and plays an efficient role in stem cell regeneration cancer and other disease states [116]. The recent revelation of its role in myocardial infarction by polarizing differentiating macrophages to the anti-inflammatory M2 phenotype is its latest pharmacological application [117]. It was shown that when LPS pretreated Murine RAW264.7 macrophages were incubated with BIO, the M1 polarized cells with high iNOS expression were diverted toward the M2 anti-inflammatory macrophage phenotype with expression of arginase-1 (Arg1). Thus, BIO maybe considered a potent M1–M2 modulator in inflammatory signaling. Bis-N-norgliovictin, a small-molecule compound from a marine fungus, has been shown to diminish M1 macrophage polarization in the liver, although its direct role in promoting the M2 phenotype is not clear [118]. 2-Amino-3H-Phenoxazin-3-One (APO) is from an extract of the edible brown mushroom *Agaricusbisporus*. APO has been shown to inhibit NO and IL-6 production in response to LPS by IFN-γ in mouse peritoneal macrophages and RAW264.7 cells respectively. It has also been shown to increase the secretion of the anti-inflammatory cytokine IL-4 in T cells, where it promotes CD4 polarization. Moreover, studies have clearly suggested the role of APO-induced polarization toward the Th2 subset, via the downregulation of IL-12 production [119]. *Cis-*palmitoleate (C16:1 n-7), a monounsaturated fatty acid, confers an anti-inflammatory M2-like polarization to macrophages by promoting anti-inflammatory gene expression (*Mrc1*, *Tgfb1*, *Il10*, and *Mgl2*) and oxidative metabolism, characteristic of M2 macrophages. It also has been shown to prevent palmitate-induced IκBα degradation, RelA nuclear translocation, NO production, and cytokine secretion [120], Besifloxacin is a novel fluoroquinolone initially developed by Bausch & Lomb for the topical treatment of ophthalmic infections [121]. It significantly inhibits LPS-stimulated cytokine production including GM-CSF, IL-1β, IL-8, IP-10, MCP-1 and MIP-1. Another compound bestatin, a dipeptide obtained from *Streptomyces olivoreticuli*, suppresses the expression of the pro-inflammatory cytokines and stimulates anti-inflammatory cytokine production by activating human monocytes [122]. The anti-rheumatic drug Chloroquine has been shown to inhibit TNF-α, IL-1 and IL-6 production in mononuclear phagocytes [123]. Pure Cell Complex (PCT)-233, an active molecular complex from the mesophyll tissue of *Spinaciaoleacea*, in combination with the corticosteroid drug budesonide, increases IL-10 production in AM [124]. Synthetic Niacin generally used as a dietary supplement has been shown to reduce the levels of TNF-α, IL-6 and IL-1β after LPS-stimulation in lung macrophages. Reports suggest the inhibition of NF-κB activation by niacin is through the blocking of NF-κB phosphorylation [125]. The fungal extracts of Cyclosporine (CsA) have been shown to inhibit LPS-mediated release of inflammatory cytokines in AMs, at concentrations as low as 2 ng ml-1 [126]. Dobutamine is a synthetic catecholamine that modulates LPS-induced MIP-1α and IL-8 production in human monocytes [127]. Acrolein (2-propenal) is a ubiquitous component of environmental pollutants as well as a natural constituent of several foods generated during inflammation or the oxidation of unsaturated lipids. It has shown to inhibit the release of IL-1β, TNF-α, and IL-12 in AM [128]). Pravastatin sodium (PSS) is a chemically complex compound and is a derivative of ML236B (compactin), identified in a fungus called *Penicilliumcitrinum.* It inhibits pro-inflammatory cytokine IL-8 production [129], particularly after thrombin treatment in human aortic endothelial cells via the inhibition of p44/42 MAPK [130]. Docosahexaenoic acid (DHA) is long-chain n-3 polyunsaturated fatty acid found in fish oil. DHA at higher concentrations may selectively decrease the pro-inflammatory cytokine production of IL-1β and TNF-α in THP-1 monocytes [131], NF-kB transcriptional activity, and upstream cytoplasmic signaling events [132]. Glatiramer acetate (GLAT) is a mixture of basic polypeptides. It enhances constitutive and LPS-induced production of IL-10 and inhibits TNF-α synthesis [133]. GLAT treatment induces a preferential Th2 deviation and also inhibits the type I IFN pathway further diverting to the M2 phenotype in monocytes [134]. Very recently, investigators have identified an important role of azithromycin in increasing anti-inflammatory and decreasing pro-inflammatory responses in macrophages. These investigators found that the antibiotic, which has long been used for a number of bacterial infections, shows anti-inflammatory activity in spinal cord injury [18]. Cilostazol and β-Ionone are synthetic compounds, shown to be anti-Inflammatory in BV2 microglial cells by suppressing the M1 specific NF-kB and MAPK activation [135–136].

## CONCLUSIONS

On exposure to external stimuli, macrophages can differentiate into pro-inflammatory (M1) or anti-inflammatory (M2) phenotypes. However, the molecular mechanisms arising from or leading to these diversions are still only partially understood. It is a common knowledge that microbial products such as LPS or Th1 cytokines including TNF and IL-6 polarize macrophages toward the M1 type thereby releasing pro-inflammatory cytokines responsible for initiating an inflammatory cascade that clears the invading microbes. In contrast, the Th2 cytokines including IL-4 and IL-13 polarize the macrophages to the M2 type, which release anti-inflammatory cytokines, thereby contributing to tissue repair and remodeling [1–2]. Recently, many breakthrough discoveries have been made in which investigators have identified therapeutically important natural compounds and molecules that have the ability to pharmacologically modulate this interconversion, particularly towards the M2 phenotype. These natural modulators include chemical entities from various classes, including those from stilbenes, polyphenols, flavonoids, terpenes, anthraquinones and various others from diverse origins. Investigators around the world have independently verified their pharmacological activities for M1–M2 polarization, with clear-cut roles as anti-inflammatory agents. Apart from the natural sources, few studies have also successfully demonstrated the pharmacological activities of compounds isolated from fungi and other microbes as well as laboratory synthesized compounds and known drugs. These miscellaneous compounds also modulate the M1 to M2 phenotypic conversion by varied pathways. In summary, the contribution of natural products as anti-inflammatory agents via the modulation of M1 and M2 phenotypes is unquestionable. Although their potent role in M1–M2 phenotypic modulation is clear, there is an unmet need for a clear-cut understanding of the exact molecular mechanisms involved in this modulation. Hence, an in-depth investigation of the molecular pathways as well as the key players involved in modulating the M1–M2 phenotypes by these agents are needed. This would pave the way not only for a better understanding of the M1–M2 phenotypic changes but would also result in the discovery of novel analogs that may be more potent in inhibiting inflammation via M1–M2 modulation.

## SUPPLEMENTARY MATERIALS TABLE




