# Supplementary material for: Phytochemicals as modulators of M1-M2 macrophages in inflammation
Source: Oncotarget. 2018 Apr 3;9(25):17937–50. doi: 10.18632/oncotarget.24788 (PMC5915167; doi:10.18632/oncotarget.24788)
Supplement: Supplementary file 1 [file oncotarget-09-17937-s001.pdf]

Phytochemicals as modulators of M1-M2 macrophages in inflammation

SUPPLEMENTARY MATERIALS

Supplementary Table 1: Pharmacological modulators of M1-M2 polarization. See Supplementary_Table_1
