# Supplementary material for: Phytochemicals as modulators of M1-M2 macrophages in inflammation
Source: Oncotarget. 2018 Apr 3;9(25):17937–50. doi: 10.18632/oncotarget.24788 (PMC5915167; doi:10.18632/oncotarget.24788)
Supplement: Supplementary file 2 [file oncotarget-09-17937-s002.docx]

**Supplementary Table 1: Pharmacological modulators of M1-M2 polarization**

| S. No. | Name | Role | Targets/ Mechanism of action | Diseases | PubChem CID | References |
| --- | --- | --- | --- | --- | --- | --- |
| 1. | Lupeol or Fargasterol | Anti-inflammatory and anti-arthritic | Inhibits the productionof pro-inflammatory cytokines like TNF-α and IL-2, IFN-ᵞ | Inflammation, cancer, arthritis, diabetes, heart diseases, renal and hepatic toxicity | 259846 https://pubchem.ncbi.nlm.nih.gov/compound/259846 | 22 |
| 2. | Resveratrol | Anti-oxidant activity | Modulation of proteins like Transthyretin, Troponin C, Sulfotransfase1B1,  Quinone reductase 2, PPAR-γ, Concanavalin A etc. | Vasodilation, platelet aggregation in atherosclerosis, neurodegeneration etc. | 445154  https://pubchem.ncbi.nlm.nih.gov/compound/445154 | 40 |
| 3. | Malibatol A | Anti-inflammatory | Protects against brain injury through reversing mitochondrial dysfunction | Brain stroke | 478623  <https://pubchem.ncbi.nlm.nih.gov/compound/478623> | 44 |
| 4. | Geraniin | Anti-tumorigenic, anti-inflammatory | Inhibits the LPS- and IFN-γ-induced expression of pro-inflammatory genes iNOS, TNF-α and IL-1β | Tumor and other inflammatory diseases | 3001497  <https://pubchem.ncbi.nlm.nih.gov/compound/3001497> | 49 |
| 5. | Compound A (CpdA) | Anti- diabetic and anti- inflammatory | Down-modulatesTNF-α induced pro-inflammatory gene expression like IL-6 and E-selectin, down-modulates NF-κB-driven genes | Diabetes and Autoimmune neuropathies | * | 55 |
| 6. | CP-25 (Paeoniflorin-6'-O-benzene sulfonate) | Anti- arthritic, prevents bone damage | Modulates inflammatory mediators like Th-17,IL-17 | Rheumatoid Arthritis | 442534  https://pubchem.ncbi.nlm.nih.gov/compound/442534 | 58 |
| 7. | Aloe-emodin (AE) | Anti- inflammatory | Blocks mRNA expression of iNOS and cyclooxygenase-2 inLPS- stimulated macrophages; inhibits the NF-κB/interferon regulatory factor 5 (IRF5)/signal transducer activator of transcription 1 (STAT1) and IRF4/STAT6 and signaling pathways; inhibits and p38 MAPK pathway | Inflammatory diseases | 10207  https://pubchem.ncbi.nlm.nih.gov/compound/10207 | 63-65 |
| 8. | Quercitin | Anti- oxidant, anti-artherogenic, anti- carcinogenic & neuroactive | Lowers pro-inflammatory cytokine production by enhancing AMPK α1 phosphorylation. Down regulates the expression of inflammatory genes: TNF-α, IL-6, IL-8, IL-1β, IP10, COX-2, phosphorylated c-Jun N-terminal kinase (JNK), c-Jun, and IκBα degradation in macrophages | Arthritis, Cancer and other inflammatory diseases | 5280343  https://pubchem.ncbi.nlm.nih.gov/compound/5280343 | 68-70 |
| 9. | Curcumin | Anti-cancer and anti-inflammatory | inhibits production of IL-8,(MIP-1α), MCP-1, IL-1 β, and TNF-αby LPS-stimulated monocytes, alveolar macrophages (AM); inhibits TNF-α and IL-1βexpression | Cancer, tendon inflammation, inflammatory diseases | 969516  https://pubchem.ncbi.nlm.nih.gov/compound/969516 | 79 |
| 10. | Naringenin | Anti- neuroinflammatory | Down regulates iNOS and COX – 2 expression, (ICAM-1), MCP-1,TNF-α and IL-6 ; induce SOCS3 expression | Colitis, Pancreatitis, neuroinflammatory diseases | 932  https://pubchem.ncbi.nlm.nih.gov/compound/932 | 86 |
| 11. | Apigenin | Suppresses obesity induced inflammation | Inhibits COX-2 and NF-ĸB expression; downregulates cytokine and NO production | Obesity related inflammation | 5280443  https://pubchem.ncbi.nlm.nih.gov/compound/5280443 | 93 |
| 12. | Chrysin | Anti-inflammatory | Activates PPARγ | Inflammatory diseases | 5281607  https://pubchem.ncbi.nlm.nih.gov/compound/5281607 | 96 |
| 13. | Procyanidin | Anti- inflammatory | Suppresses MAPK and NF- ĸB pathway | Inflammatory diseases | 107876  https://pubchem.ncbi.nlm.nih.gov/compound/107876 | 97 |
| 14. | Epigallocatechin gallate (EGCG) | Anti- inflammatory | Inhibits iNos and COX-2 expression | Inflammatory diseases | 65064  https://pubchem.ncbi.nlm.nih.gov/compound/65064 | 98 |
| 15. | Berberine (BBR) | Anti- oxidant | Negatively regulates NF-ĸB pathway; down regulatesTh1 & Th17 cytokine secretion | Inflammatory diseases | 2353  https://pubchem.ncbi.nlm.nih.gov/compound/2353 | 103 |
| 16. | Apocynin | Anti- inflammatory | Inhibits NF-κB activation; inhibits production of TNF-α, IL-1β and IL-6 | Inflammation in lungs and other tissues | 9804654  https://pubchem.ncbi.nlm.nih.gov/compound/9804654 | 105 |
| 17. | Paeonol | Anti- inflammatory | Inhibits the pro-inflammatory TNF-α and IL-1β production; anti-inflammatory IL-10 production | Neurodegenerative diseases and arthritis | 11092  https://pubchem.ncbi.nlm.nih.gov/compound/11092 | 107 |
| 18. | Forskolin (coleonol) | Anti- oxidant, anti-inflammatory,anti- diabetic | Strongly inhibit the LPS-induced increase in MCP-1, TLR-4, and NFκB1 mRNA levels in adipocytes | Diabetes and other inflammatory diseases | 47936  https://pubchem.ncbi.nlm.nih.gov/compound/47936 | 109 |
| 19. | Triptolide (diterpenoid epoxide) | Anti carcinogenic, anti- inflammatory | Inhibit the LPS-induced expression of pro-inflammatory cytokines and chemokines likeIL-6,G-CSF, MCP-1, IL-8, ICAM-1 | Pancreatic cancer, polycystic kidneys in mouse | 107985  https://pubchem.ncbi.nlm.nih.gov/compound/107985 | 110 |
| 20. | Terpinen-4-ol | Anti- bacterial, anti-fungal, anti-inflammatory | Suppresses the production of inflammatory mediators like TNF-α, IL-1β, IL-8, IL-10 and PGE2 | Bacterial and fungal infections and inflammatory diseases | 11230  https://pubchem.ncbi.nlm.nih.gov/compound/11230 | 112-113 |
| 21. | Dexamethasone | Anti-allergic, anti rheumatic, anti –inflammatory and immunosuppressant | Suppresses M1 and supports M2 polarization | Allergies, asthama, rheumatic problems, skin diseases, brain swelling etc. | 5743  https://pubchem.ncbi.nlm.nih.gov/compound/5743 | 114 |
| 22. | Fucoidan | Anti-inflammatory | Suppresses pro-inflammatory mediators like NO, PGE₂, iNOS, COX-2, MCP-1, IL-1β and TNF-α | Inflammatory diseases | 92023653  https://pubchem.ncbi.nlm.nih.gov/compound/92023653 | 115 |
| 23. | BIO (6-bromoindirubin-3′-oxime) | Anti-inflammatory, anti-carcinogenic | a potent M1-M2 modulator; | Cancer and other inflammatory diseases | 5287844  https://pubchem.ncbi.nlm.nih.gov/compound/5287844 | 116 |
| 26. | cis-palmitoleate | Anti- inflammatory | promoting anti-inflammatory gene expression (Mrc1, Tgfb1, Il10, Mgl2) and oxidative metabolism | Myocardial infarction | 445638  https://pubchem.ncbi.nlm.nih.gov/compound/445638 | 120 |
| 27. | Besifloxacin | Anti- inflammatory | Inhibits LPS-stimulated cytokine production including GM-CSF, IL-1β, IL-8, IP-10, MCP-1 and MIP-1α | Ophthalmic infection | 10178705  https://pubchem.ncbi.nlm.nih.gov/compound/10178705 | 121 |
| 28. | Bestatin | Anti-inflammatory | Stimulates the anti-inflammatory cytokine production by activated human monocytes | Inflammatory diseases | 72172  https://pubchem.ncbi.nlm.nih.gov/compound/72172 | 122 |
| 29. | Chloroquine | Anti- rheumatic | TNF- α, IL-1 and IL-6 production | Rheumatic and other inflammatory diseases | 2719  https://pubchem.ncbi.nlm.nih.gov/compound/2719 | 123 |
| 30. | Pure Cell Complex (PCT)-233 | Anti-inflammatory | Increases IL-10 production | Inflammatory diseases | * | 124 |
| 31. | Niacin | Anti-inflammatory | Reduces the levels of TNF-α, IL-6 and IL-1β after LPS-stimulation in lung macrophages; blocks NF-κB phosphorylation | Inflammatory diseases | 938  https://pubchem.ncbi.nlm.nih.gov/compound/938 | 125 |
| 32. | Cyclosporine (CsA) | Anti-inflammatory | Inhibits LPS-mediated release of inflammatory cytokines | Inflammatory diseases | 5284373  https://pubchem.ncbi.nlm.nih.gov/compound/5284373 | 126 |
| 33. | Dobutamine | Anti-inflammatory | Modulates LPS-induced MIP-1α and IL-8 production in human monocytes | Inflammatory diseases | 36811  https://pubchem.ncbi.nlm.nih.gov/compound/36811 | 127 |
| 34. | Acrolein | Anti-inflammatory | inhibits the release of IL-1beta, TNF-alpha, and IL-12 | Inflammatory diseases | 7847  https://pubchem.ncbi.nlm.nih.gov/compound/7847 | 128 |
| 35. | Pravastatin sodium (PSS) | Anti-inflammatory | Inhibits pro-inflammatory cytokine IL-8 production | Inflammatory diseases | 16759173  https://pubchem.ncbi.nlm.nih.gov/compound/16759173 | 129 |
| 36. | Glatiramer acetate(GLAT) | Anti-inflammatory | Enhances constitutive and LPS-induced production of IL-10and inhibits TNF-α synthesis | Inflammatory diseases | 3081884  https://pubchem.ncbi.nlm.nih.gov/compound/3081884 | 133 |
| 37. | Azithromycin | Anti-inflammatory | Increasies anti-inflammatory and decreases pro-inflammatory responses in macrophages | Inflammatory diseases | 447043  https://pubchem.ncbi.nlm.nih.gov/compound/447043 | 18 |
| 39. | Cilostazol | Anti-inflammatory | Suppresses the M1 specific NF-kB and MAPK activation | Inflammatory diseases | 2754  https://pubchem.ncbi.nlm.nih.gov/compound/2754 | 135 |
| 40. | β-Ionone | Anti-inflammatory | Suppresses the M1 specific NF-kB and MAPK activation | Inflammatory diseases | 638014  https://pubchem.ncbi.nlm.nih.gov/compound/638014 | 136 |
| 41. | Bis-N-norgliovictin | Anti-inflammatory | Diminishes M1 [macrophage](http://topics.sciencedirect.com/topics/page/Macrophages) polarization | Inflammatory diseases | * | 118 |
| 42. | 2-amino-3H-phenoxazin-3-one (APO) | Anti-inflammatory | Inhibits NO and IL-6 production in response to LPS by IFN-ᵞ | Inflammatory diseases | 72725  https://pubchem.ncbi.nlm.nih.gov/compound/72725#section=Top | 119 |

*PubChem info not available
